# Supplementary material for: Modeling glioblastoma heterogeneity as a dynamic network of cell states
Source: Mol Syst Biol. 2021 Sep 16;17(9):e10105. doi: 10.15252/msb.202010105 (PMC8444284; doi:10.15252/msb.202010105)
Supplement: Supplementary file 6 — Source Data for Figure 5 [file MSB-17-e10105-s004.zip › Figure5A_sourcedata/GSEA_3017/nosigdb_stateF.GseaPreranked.1622795015644/FELDMAN_2020_G1.html]

Details for gene set FELDMAN\_2020\_G1[GSEA]

|  || Dataset | state33017 |
| Phenotype | NoPhenotypeAvailable |
| Upregulated in class | na\_neg |
| GeneSet | FELDMAN\_2020\_G1 |
| Enrichment Score (ES) | -0.8453638 |
| Normalized Enrichment Score (NES) | -5.808007 |
| Nominal p-value | 0.0 |
| FDR q-value | 0.0 |
| FWER p-Value | 0.0 |
Table: GSEA Results Summary

  

Fig 1: Enrichment plot: FELDMAN\_2020\_G1      
 Profile of the Running ES Score & Positions of GeneSet Members on the Rank Ordered List

  

| PROBE | GENE SYMBOL | GENE\_TITLE | RANK IN GENE LIST | RANK METRIC SCORE | RUNNING ES | CORE ENRICHMENT || 1 | S100B |  |  | 20 | 1.167 | 0.0007 | No |
| 2 | MALAT1 |  |  | 90 | 0.773 | -0.0251 | No |
| 3 | MIAT |  |  | 263 | 0.542 | -0.1015 | No |
| 4 | SCG2 |  |  | 354 | 0.485 | -0.1397 | No |
| 5 | PRCP |  |  | 426 | 0.460 | -0.1692 | No |
| 6 | IGFBP5 |  |  | 564 | 0.410 | -0.2303 | No |
| 7 | DLG1 |  |  | 1013 | 0.307 | -0.4388 | No |
| 8 | SCRG1 |  |  | 1032 | 0.304 | -0.4447 | No |
| 9 | HNRNPA2B1 |  |  | 1503 | -0.261 | -0.6640 | No |
| 10 | HSP90AA1 |  |  | 1535 | -0.268 | -0.6763 | No |
| 11 | PSIP1 |  |  | 1564 | -0.275 | -0.6871 | No |
| 12 | RAN |  |  | 1772 | -0.350 | -0.7817 | No |
| 13 | ILF2 |  |  | 1795 | -0.360 | -0.7889 | No |
| 14 | C12orf75 |  |  | 1832 | -0.380 | -0.8026 | No |
| 15 | EXOSC8 |  |  | 1865 | -0.397 | -0.8142 | No |
| 16 | GINS2 |  |  | 1932 | -0.445 | -0.8415 | Yes |
| 17 | MZT1 |  |  | 1936 | -0.447 | -0.8390 | Yes |
| 18 | SNRPB |  |  | 1937 | -0.447 | -0.8352 | Yes |
| 19 | HNRNPDL |  |  | 1940 | -0.448 | -0.8322 | Yes |
| 20 | DDX39A |  |  | 1957 | -0.458 | -0.8358 | Yes |
| 21 | DNMT1 |  |  | 1969 | -0.465 | -0.8370 | Yes |
| 22 | PTGES3 |  |  | 1970 | -0.467 | -0.8329 | Yes |
| 23 | CMC2 |  |  | 1977 | -0.473 | -0.8316 | Yes |
| 24 | ARL6IP1 |  |  | 2001 | -0.494 | -0.8382 | Yes |
| 25 | TK1 |  |  | 2003 | -0.497 | -0.8343 | Yes |
| 26 | SIVA1 |  |  | 2014 | -0.505 | -0.8347 | Yes |
| 27 | FEN1 |  |  | 2018 | -0.511 | -0.8317 | Yes |
| 28 | RRM1 |  |  | 2023 | -0.516 | -0.8291 | Yes |
| 29 | RANBP1 |  |  | 2024 | -0.517 | -0.8246 | Yes |
| 30 | CDCA3 |  |  | 2041 | -0.529 | -0.8276 | Yes |
| 31 | TROAP |  |  | 2046 | -0.533 | -0.8248 | Yes |
| 32 | KIF22 |  |  | 2053 | -0.541 | -0.8230 | Yes |
| 33 | SRSF7 |  |  | 2056 | -0.544 | -0.8192 | Yes |
| 34 | STMN1 |  |  | 2063 | -0.567 | -0.8171 | Yes |
| 35 | PTMA |  |  | 2065 | -0.570 | -0.8126 | Yes |
| 36 | ZWINT |  |  | 2069 | -0.577 | -0.8090 | Yes |
| 37 | CENPA |  |  | 2073 | -0.586 | -0.8054 | Yes |
| 38 | BUB3 |  |  | 2078 | -0.596 | -0.8021 | Yes |
| 39 | AURKB |  |  | 2079 | -0.598 | -0.7969 | Yes |
| 40 | GGH |  |  | 2080 | -0.603 | -0.7917 | Yes |
| 41 | CENPK |  |  | 2082 | -0.607 | -0.7869 | Yes |
| 42 | USP1 |  |  | 2084 | -0.612 | -0.7820 | Yes |
| 43 | KNSTRN |  |  | 2085 | -0.615 | -0.7767 | Yes |
| 44 | PLK1 |  |  | 2087 | -0.624 | -0.7717 | Yes |
| 45 | MIS18A |  |  | 2096 | -0.639 | -0.7700 | Yes |
| 46 | DNAJC9 |  |  | 2097 | -0.641 | -0.7644 | Yes |
| 47 | PHF19 |  |  | 2101 | -0.650 | -0.7602 | Yes |
| 48 | SNRPD1 |  |  | 2105 | -0.653 | -0.7559 | Yes |
| 49 | CKAP2L |  |  | 2109 | -0.663 | -0.7516 | Yes |
| 50 | AURKA |  |  | 2110 | -0.673 | -0.7458 | Yes |
| 51 | RPA3 |  |  | 2112 | -0.678 | -0.7404 | Yes |
| 52 | LSM3 |  |  | 2114 | -0.694 | -0.7348 | Yes |
| 53 | CKS2 |  |  | 2115 | -0.694 | -0.7288 | Yes |
| 54 | CDC20 |  |  | 2117 | -0.695 | -0.7232 | Yes |
| 55 | RAD21 |  |  | 2118 | -0.697 | -0.7172 | Yes |
| 56 | ANP32B |  |  | 2119 | -0.700 | -0.7111 | Yes |
| 57 | CDCA2 |  |  | 2120 | -0.705 | -0.7050 | Yes |
| 58 | CENPM |  |  | 2125 | -0.709 | -0.7007 | Yes |
| 59 | CKAP2 |  |  | 2131 | -0.729 | -0.6968 | Yes |
| 60 | VRK1 |  |  | 2134 | -0.743 | -0.6913 | Yes |
| 61 | NDC80 |  |  | 2142 | -0.775 | -0.6878 | Yes |
| 62 | H2AFV |  |  | 2143 | -0.779 | -0.6811 | Yes |
| 63 | FBXO5 |  |  | 2144 | -0.779 | -0.6743 | Yes |
| 64 | KIF23 |  |  | 2145 | -0.782 | -0.6676 | Yes |
| 65 | GMNN |  |  | 2146 | -0.785 | -0.6607 | Yes |
| 66 | ANLN |  |  | 2149 | -0.797 | -0.6548 | Yes |
| 67 | DEPDC1 |  |  | 2152 | -0.809 | -0.6487 | Yes |
| 68 | DEK |  |  | 2154 | -0.817 | -0.6421 | Yes |
| 69 | DUT |  |  | 2155 | -0.824 | -0.6349 | Yes |
| 70 | NUCKS1 |  |  | 2158 | -0.832 | -0.6287 | Yes |
| 71 | LSM5 |  |  | 2159 | -0.835 | -0.6214 | Yes |
| 72 | MCM7 |  |  | 2160 | -0.839 | -0.6142 | Yes |
| 73 | TUBB4B |  |  | 2162 | -0.843 | -0.6073 | Yes |
| 74 | HMGB3 |  |  | 2164 | -0.852 | -0.6004 | Yes |
| 75 | KIF20B |  |  | 2165 | -0.852 | -0.5930 | Yes |
| 76 | DTYMK |  |  | 2168 | -0.858 | -0.5865 | Yes |
| 77 | NUF2 |  |  | 2176 | -0.877 | -0.5822 | Yes |
| 78 | CEP55 |  |  | 2177 | -0.883 | -0.5745 | Yes |
| 79 | KPNA2 |  |  | 2178 | -0.894 | -0.5668 | Yes |
| 80 | CCDC34 |  |  | 2180 | -0.898 | -0.5595 | Yes |
| 81 | CCNA2 |  |  | 2181 | -0.902 | -0.5516 | Yes |
| 82 | KIF4A |  |  | 2186 | -0.921 | -0.5455 | Yes |
| 83 | ORC6 |  |  | 2188 | -0.937 | -0.5379 | Yes |
| 84 | CENPH |  |  | 2191 | -0.943 | -0.5307 | Yes |
| 85 | CENPW |  |  | 2192 | -0.943 | -0.5225 | Yes |
| 86 | TUBA1B |  |  | 2193 | -0.947 | -0.5143 | Yes |
| 87 | LMNB1 |  |  | 2194 | -0.950 | -0.5060 | Yes |
| 88 | UBE2T |  |  | 2196 | -0.965 | -0.4981 | Yes |
| 89 | CDKN3 |  |  | 2199 | -0.979 | -0.4906 | Yes |
| 90 | CCNB2 |  |  | 2200 | -0.983 | -0.4821 | Yes |
| 91 | CKS1B |  |  | 2201 | -0.987 | -0.4735 | Yes |
| 92 | HIST1H1E |  |  | 2202 | -0.992 | -0.4649 | Yes |
| 93 | ESCO2 |  |  | 2204 | -1.004 | -0.4566 | Yes |
| 94 | SMC2 |  |  | 2205 | -1.006 | -0.4479 | Yes |
| 95 | SPC25 |  |  | 2208 | -1.019 | -0.4400 | Yes |
| 96 | CENPE |  |  | 2211 | -1.036 | -0.4320 | Yes |
| 97 | DHFR |  |  | 2212 | -1.040 | -0.4230 | Yes |
| 98 | NUDT1 |  |  | 2214 | -1.054 | -0.4143 | Yes |
| 99 | CLSPN |  |  | 2216 | -1.060 | -0.4056 | Yes |
| 100 | CENPN |  |  | 2217 | -1.079 | -0.3962 | Yes |
| 101 | UBE2C |  |  | 2218 | -1.081 | -0.3868 | Yes |
| 102 | CENPU |  |  | 2219 | -1.088 | -0.3774 | Yes |
| 103 | H2AFZ |  |  | 2220 | -1.090 | -0.3679 | Yes |
| 104 | HMGB1 |  |  | 2221 | -1.116 | -0.3583 | Yes |
| 105 | NCAPG |  |  | 2222 | -1.122 | -0.3485 | Yes |
| 106 | H2AFX |  |  | 2223 | -1.146 | -0.3386 | Yes |
| 107 | CCNB1 |  |  | 2224 | -1.148 | -0.3286 | Yes |
| 108 | CDK1 |  |  | 2225 | -1.153 | -0.3186 | Yes |
| 109 | PTTG1 |  |  | 2226 | -1.198 | -0.3082 | Yes |
| 110 | ANP32E |  |  | 2227 | -1.200 | -0.2978 | Yes |
| 111 | MAD2L1 |  |  | 2228 | -1.204 | -0.2874 | Yes |
| 112 | UBE2S |  |  | 2229 | -1.205 | -0.2769 | Yes |
| 113 | GTSE1 |  |  | 2230 | -1.220 | -0.2663 | Yes |
| 114 | DBF4 |  |  | 2232 | -1.247 | -0.2560 | Yes |
| 115 | HMGN2 |  |  | 2233 | -1.267 | -0.2450 | Yes |
| 116 | RRM2 |  |  | 2234 | -1.268 | -0.2340 | Yes |
| 117 | DLGAP5 |  |  | 2235 | -1.273 | -0.2230 | Yes |
| 118 | SMC4 |  |  | 2236 | -1.281 | -0.2118 | Yes |
| 119 | PBK |  |  | 2237 | -1.325 | -0.2004 | Yes |
| 120 | ATAD2 |  |  | 2238 | -1.381 | -0.1884 | Yes |
| 121 | TMPO |  |  | 2239 | -1.382 | -0.1764 | Yes |
| 122 | PRC1 |  |  | 2240 | -1.397 | -0.1643 | Yes |
| 123 | HMGB2 |  |  | 2241 | -1.469 | -0.1515 | Yes |
| 124 | BIRC5 |  |  | 2242 | -1.469 | -0.1388 | Yes |
| 125 | HIST1H1C |  |  | 2243 | -1.485 | -0.1259 | Yes |
| 126 | ASPM |  |  | 2244 | -1.490 | -0.1130 | Yes |
| 127 | TOP2A |  |  | 2246 | -1.562 | -0.0999 | Yes |
| 128 | NUSAP1 |  |  | 2247 | -1.600 | -0.0860 | Yes |
| 129 | TPX2 |  |  | 2248 | -1.609 | -0.0721 | Yes |
| 130 | CENPF |  |  | 2249 | -1.691 | -0.0574 | Yes |
| 131 | HIST1H1B |  |  | 2251 | -1.796 | -0.0423 | Yes |
| 132 | MKI67 |  |  | 2252 | -1.892 | -0.0259 | Yes |
| 133 | HIST1H4C |  |  | 2253 | -2.984 | -0.0000 | Yes |
Table: GSEA details [plain text format]

  

Fig 2: FELDMAN\_2020\_G1: Random ES distribution      
 Gene set null distribution of ES for **FELDMAN\_2020\_G1**
